# Case report: Malignant teratoma of the uterine corpus

**DOI:** 10.1186/1471-2407-9-195

**Published:** 2009-06-18

**Authors:** Thomas Newsom-Davis, Daniel Poulter, Rebecca Gray, Mohammed Ameen, Iain Lindsay, Kyriakos Papanikolaou, Simon Butler-Manuel, Timothy Christmas, Peter Townsend, Michael Seckl

**Affiliations:** 1 Department of Medical Oncology, Imperial College School of Medicine, Charing Cross Hospital, Fulham Palace Road, London, UK; 2 Department of Obstetrics and Gynaecology, East Surrey Hospital, Redhill, Surrey, UK; 3 Department of Medicine, East Surrey Hospital, Redhill, Surrey, UK; 4 Derpartment of Histopathology, East Surrey Hospital, Redhill, Surrey, UK; 5 Department of Histopathology, Imperial College School of Medicine, Charing Cross Hospital, Fulham Palace Road, London, UK; 6 Department of Urological Surgery, Imperial College School of Medicine, Charing Cross Hospital, Fulham Palace Road, London, UK

## Abstract

**Background:**

Teratomas are the commonest germ cell tumours and are most frequently found in the testes and ovary. Extragonadal teratomas are rare and mainly occur in midline structures. Uterine teratomas are extremely rare with only a few previous case reports, usually involving mature teratomas of the uterine cervix.

**Case Presentation:**

We report an 82-year-old lady presenting with post-menopausal bleeding. Initial investigations revealed a benign teratoma of the uterus which was removed. Her symptoms persisted and a recurrent, now malignant, teratoma of the uterine corpus was resected at hysterectomy. Six months after surgery she relapsed with para-aortic lymphadenopathy and was treated with a taxane, etoposide and cisplatin-containing chemotherapy regimen followed by retroperitoneal lymph node dissection.

**Conclusion:**

In this report we discuss the aetiology, diagnosis and management of uterine teratomas, and review previous case studies.

## Background

Teratomas (from the Greek *teraton *for monster) are the most common germ cell tumours (GCTs) and are composed of two or more germ layers (ectoderm, mesoderm or endoderm), derived from a pluripotential malignant precursor cell. Mature teratomas consist of adult-type differentiated components such as cartilage and glandular epithelium. Immature teratomas contain tissue with partial somatic differentiation similar to that in foetal tissue. Teratomas with malignant transformation demonstrate aggressive neoplastic growth of one or more of the histological components.

In adults, teratomas most commonly arise in the testes or more rarely the ovaries, with the peak age of presentation between 20 and 40 years [1]. However extragonadal teratomas can occur and are found in any mid-line structure including the thyroid, retroperitoneum, mediastinum, pericardium and brain (typically the pituitary and/or pineal gland). Very rarely, teratomas are found in other solid (e.g. breast, parotid gland, liver) and hollow (e.g. oesophagus, stomach, bladder, uterine cervix) organs. Here, we report a case of uterine teratoma, which is a very rare presentation of this tumour.

## Case presentation

An 82-year-old lady was referred to gynaecology outpatients in June 2007 with a one month history of post menopausal bleeding. Her past gynaecological history included a negative hysteroscopy in 1998, and previous use of hormone replacement therapy. She had previously given birth to two children. The patient was fit and well, with no significant past medical history apart from hypertension for which she took bendroflumethiazide and atenolol.

**Figure 1:**
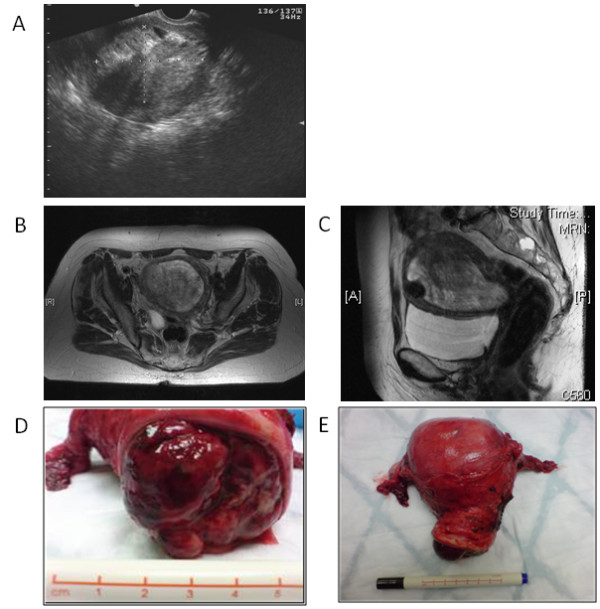
**Radiological and surgical specimen appearances of uterine teratoma**.

Physical examination revealed a bulky uterus with no adnexal masses. A pipelle biopsy demonstrated only tiny fragments of blood clot. A subsequent transvaginal ultrasound scan showed a large endometrial mass with calcification (Figure 1A). The ovaries appeared normal. She underwent a hysteroscopy in July 2007 when a 6 cm uterine fibrotic polyp, which filled the uterine cavity, was removed.

Microscopy demonstrated polypoid tissue with a variably cellular and fibrotic stroma, focal adipose and possible chondroid metaplasia, but no malignant features. The glands showed focal mucinous and keratinising sqaumous epithelial metaplasia. There was focal nuclear atypia, focal mitotic activity and occasional cribriform gland fusion. These features were in keeping with either atypical complex hyperplasia within an endometrial polyp associated with metaplastic changes, or a polypoid uterine teratoma.

Immunohistochemistry showed positive staining of the small crowded epithelium for the epithelial marker cytokeratin (CK)-7 and the thyroid and lung marker TTFI. There was positive staining of the chondroid area for S100 protein, focal staining of dilated gland epithelium and stromal cells for oestrogen receptor and progesterone receptor, and staining of stromal cells for smooth muscle α-actin (SMA). Thyroglobulin, desmin, CK20 and CDX2 staining was negative. A diagnosis of benign teratoma with thyroid gland and cartilaginous elements was therefore made.

Following hysteroscopy, the bleeding continued. A repeat ultrasound scan revealed that the teratoma had grown back almost completely filling the uterine cavity. A magnetic resonance imaging (MRI) scan in November 2007 showed the tumour filling and distending the endometrial cavity and extending down into the cervix (Figure 1B–C). There was evidence of posterior wall myometrial invasion but there was no lymphadenopathy and the ovaries appeared normal. Tumour markers including alpha-fetoprotein (AFP), carcinoembryonic antigen (CEA) and Ca19-9 were within normal limits. Serum Ca125 was slightly elevated at 42 U/ml (normal range 0–35 units (U)/ml) and lactate dehydrogenase (LDH) raised at 372 IU/L (normal range 125–250 U/ml).

The patient proceeded to a total abdominal hysterectomy and bilateral salpingo-oophorectmy in December 2007. At operation, the uterus was found to contain a haemorrhagic polypoid tumour (110 × 80 × 70 mm) arising from the posterior aspect of the endometrial cavity (Figure 1D–E). Uterine size was equivalent to that of a 12-week gestation uterus.

**Figure 2:**
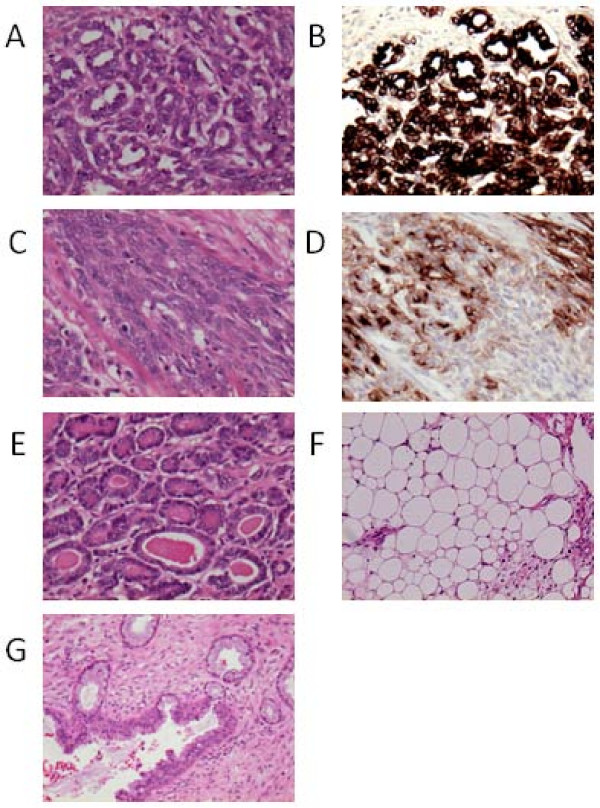
**Histopathological appearances of malignant teratoma of the uterus**.

Microscopically the tumour was a teratoma containing mature and immature elements with mixed malignant transformation (Figure 2). The tissue types found included squamous and glandular epithelium, thyroid parenchyma, smooth muscle, connective and adipose tissue. In addition there were areas of immature bone, invasive adenocarcinoma, and papillary thyroid carcinoma. There was extensive lymphovascular invasion and deep myometrial, but not serosal, involvement. The omentum, cervix, fallopian tubes and ovaries were free of tumour. Immunohistochemistry showed that the malignant epithelial components were positive for CK-7 and TTF-1, but negative for CK20 and thyroglobulin. One area of the tumour stained positive for desmin but not for SMA, S100 or CD10, suggesting that this is likely to be a small focus of myogenic sarcoma.

The histopathological conclusion was of a poorly differentiated adenocarcinoma and a focal myogenic sarcoma arising in a polypoid uterine teratoma with mature and immature elements. A post-operative computer tomography (CT) scan of the thorax, abdomen and pelvis found no evidence of distant disease giving an overall International Federation of Gynaecology and Obstetrics (FIGO) disease stage 1C.

The patient recovered well from surgery and was referred for oncological follow up. Given her age and performance status a surveillance approach was taken with regular clinical examinations, serial tumour markers and routine CT scans. Initially in remission, six months post-operatively para-aortic lymphadenopathy was detected on CT although she remained asymptomatic with an Eastern Cooperative Oncology Group (ECOG) performance status of 0. In view of her age and wishes for a treatment with acceptable toxicity, the patient was commenced on an initial dose of cisplatin (20 mg/m^2^) and etoposide (100 mg/m^2^). This was well tolerated so one week later treatment was continued with a fortnightly alternating regimen of paclitaxel (135 mg/m^2^) and etoposide (150 mg/m^2^), followed by paciltaxel (135 mg/m^2^) and cisplatin (60 mg/m^2^). This treatment was chosen based on our experience of its effectiveness and tolerability in the treatment of relapsed germ cell tumours and gestational trophoblastic disease [2,3].

After three cycles of chemotherapy there was a reduction in the size of the para-aortic mass, but an increase in the cystic component suggesting possible differentiation towards a mature teratoma. Consequently she underwent a retro-peritoneal lymph node dissection in October 2008. Histology from this confirmed the presence of metastatic teratoma. Unfortunately she had a turbulent post-operative course and, although she recovered well enough to return home a month later, she sadly died shortly thereafter.

## Conclusion

Extra-gonadal GCTs represent 1%–2% of all germ cell tumours [4]. Their histological features are analogous to those of gonadal GCTs but they occur in locations other than the gonads. Extra-gonadal GCTs usually arise from midline structures, the commonest sites being the retroperitoneum and mediastinum. Teratomas arising from the uterus are extremely rare: Since the first description of a uterine teratoma by Mann in 1929 there have been just a handful of case reports [5]. The majority of these are mature teratomas and there are only three previous reports in the literature of immature teratomas arising from the uterus [6-8]. All previous reports have been in women of reproductive age and to our knowledge this is the first report of a teratoma in a post-menopausal lady. In addition this is the first report of a malignant GCT arising from a benign teratoma. A malignant teratoma arising from a dermoid in any location in the body is in itself a rare phenomenon, estimated to occur in only 0.5% of cases.

Teratomas of the uterus should be differentiated from other tumours that can present with more than one germ layer, such as a mixed Müllerian tumour, and from perforated ovarian teratomas. The differential diagnosis of mature teratoma also includes neuroectodermal tumours.

There are two hypotheses regarding the origin of uterine teratomas. The blastomere theory proposes that they arise from residual pluripotent embryonic cells left behind following a missed abortion or undelivered papyraceous twin. Foetal tissue could also be inadvertently implanted into the uterine wall during instrumentation [9,10]. This has been largely discredited however as teratoma cells have a 46,XX karyotype meaning that they are derived solely from the host and have completed meiosis [11]. Furthermore, in this case, the patient was post-menopausal, had a normal hysteroscopy in 1998, and there was no lymphoid proliferation within the teratoma. A more likely aetiology, therefore, is the parthenogenic theory which suggests that uterine teratomas arise from primordial cells that have been misplaced during ontogeny instead of migrating, as planned, from the yolk sac endoderm to the gonadal ridge [12]. The propensity of these tumours to arise in midline structures is explained by the passage of primordial cells in the midline.

Uterine teratomas usually present with abnormal per vaginal (p.v.) bleeding, p.v. discharge or pelvic pain. Although most ovarian germ cell tumours are diagnosed pre-operatively by ultrasound examination, in none of the previous reports of uterine teratomas was a correct pre-operative diagnosis made. Most were misdiagnosed as uterine or cervical polyps. This reflects the variable sonographic appearances of uterine teratomas which frequently lack the characteristic dense echogenic tubercle (Rokitansky nodule) projecting into a cystic lumen, the diffusely echogenic mass representing the presence of hair or sebaceous elements, and multiple thin, echogenic bands of hair within the cavity [12]. MRI and CT have been reported to show a cystic tumour with adipose tissue in the case of mature teratomas and a solid tumour with small foci of calcification with immature teratomas [13].

Teratomas of the uterine corpus have been treated by tumour resection or total abdominal hysterectomy, with or without pelvic and/or para-aortic lymphadenectomy [4]. Those of the uterine cervix can also be managed by conization. The role of adjuvant chemotherapy for completely resected uterine teratomas is not clear. Using ovarian GCTs as an example, some institutions advocate the use of adjuvant BEP (bleomycin, etoposide, cisplatin) chemotherapy whereas others prefer to take a surveillance approach [14]. Given the lack of experience in managing uterine teratoma patients, either can be justified. Where there is concern regarding the patient's performance status or age, as is the situation in this case, an expectant approach is not unreasonable with close clinical surveillance, regular cross-sectional imaging and monitoring of serum tumour markers (AFP, HCG, Ca125 and LDH). Due to the small number of reported cases, the prognosis of uterine teratomas is uncertain.

Although rare, uterine teratomas should be considered in the differential diagnosis of any intrauterine mass, even in the absence of typical radiological features.

## Abbreviations

AFP: Alpha-fetoprotein; CEA: Carcinoembryonic antigen; CK: Cytokeratin; CT: Computerised tomography; ECOG: Eastern Cooperative Oncology Group; FIGO: International Federation of Obstetrics and Gynaecology; GCT: Germ cell tumour; LDH: Lactate dehydrogenase; MRI: Magnetic resonance imaging; p.v.: Per vagina

## Competing interests

The authors declare that they have no competing interests.

## Authors' contributions

TND, RG and DP are the main authors. KP, SBM, TC, MS and PT were all involved in patient management and provided critical review of the manuscript. IL and MA provided images/figures.

## Consent

Written consent was obtained from the patient for publication of their details.

## Pre-publication history

The pre-publication history for this paper can be accessed here:

http://www.biomedcentral.com/1471-2407/9/195/prepub
